# Supplementary material for: Dynamic transcriptomic profiles of zebrafish gills in response to zinc supplementation
Source: BMC Genomics. 2010 Oct 11;11:553. doi: 10.1186/1471-2164-11-553 (PMC3091702; doi:10.1186/1471-2164-11-553)
Supplement: Additional file 2 — Interactive Direct Interaction Network representing the molecular interactions between zinc, copper, iron, calcium and proteins encoded by transcripts changed by zinc supplementation. Mini web-site containing index.html and hyperlinked pages in subdirectory describing a Direct Interaction Network automatically generated based on curated interactions contained within the proprietary PathwayArchitect database. Ovals represent proteins and the circles symbolize metal ions. Objects are coloured by their abundance in zebrafish at the time-point they were significantly different from the control is a scale from -4 fold (dark green) to +4 fold (dark red). Where significant differences were found at more than one time-point, the colour overlay shows expression at the first instance. Dark blue squares denote 'binding', and light blue squares 'expression'; green squares stand for 'regulation', green diamonds for 'metabolism', and green circles for 'promoter binding'. Arrow heads indicate directionality of the interaction where annotated. All nodes and edges can be further interrogated by selecting the relative area of the image. [file 1471-2164-11-553-S2.zip › PathwayArchitect Zn xs DIN/96948.html]

# PROTEIN: CRH

|  |  |
| --- | --- |
| Name | CRH |
| Type | PROTEIN |
| Description | corticotropin releasing hormone |
| Note | Corticotropin-releasing hormone (CRH) is a 41-amino acid peptide derived from a 191-amino acid preprohormone. CRH is secreted by the paraventricular nucleus (PVN) of the hypothalamus in response to stress. Marked reduction in CRH has been observed in association with Alzheimer disease and autosomal recessive hypothalamic corticotropin dificiency has multiple and potentially fatal metabolic consequences including hypoglycemia and hepatitis. In addition to production in the hypothalamus, CRH is also synthesized in peripheral tissues, such as T lymphocytes and is highly expressed in the placenta. In the placenta CRH is a marker that determines the length of gestation and the timing of parturition and delivery. A rapid increase in circulating levels of CRH occurs at the onset of parturition, suggesting that, in addition to its metabolic functions, CRH may act as a trigger for parturition. |
| Alias | Gm1347 |
|  | Corticotropin-releasing hormone |
|  | CRF |
|  | corticoliberin |
|  | corticotropin releasing factor |
|  | Crh |
|  | CRH |
|  | Corticotropin-releasing factor |


---

|  |  |
| --- | --- |
| GO Component | extracellular space |
|  | extracellular region |
|  | soluble fraction |


---

|  |  |
| --- | --- |
| GO ID | GO:0005615 |
|  | GO:0006950 |
|  | GO:0005102 |
|  | GO:0005184 |
|  | GO:0007616 |
|  | GO:0005179 |
|  | GO:0005576 |
|  | GO:0007611 |
|  | GO:0007565 |
|  | GO:0007165 |
|  | GO:0005625 |
|  | GO:0006954 |
|  | GO:0007567 |
|  | GO:0008628 |
|  | GO:0007268 |
|  | GO:0006955 |
|  | GO:0017045 |


---

|  |  |
| --- | --- |
| MIM | MIM:201400 |
|  | MIM:122560 |


---

|  |  |
| --- | --- |
| Connectivity | 2030 |


---

|  |  |
| --- | --- |
| Entrez ID | 1392 |
|  | 12918 |
|  | 81648 |


---

|  |  |
| --- | --- |
| Agilent ID | A\_43\_P12478 |
|  | A\_53\_P157511 |
|  | A\_53\_P157900 |
|  | A\_53\_P128571 |
|  | A\_23\_P31755 |
|  | A\_14\_P117657 |
|  | A\_53\_P122310 |
|  | A\_14\_P117921 |
|  | A\_14\_P201263 |


---

|  |  |
| --- | --- |
| Cellular Localization | Extracellular region |
|  | Cell |


---

|  |  |
| --- | --- |
| DbXref | KEGG pathway##04080##Neuroactive ligand-receptor interaction##http://www.genome.jp/dbget-bin/show\_pathway?rno04080+81648 |
|  | KEGG pathway##04080##Neuroactive ligand-receptor interaction##http://www.genome.jp/dbget-bin/show\_pathway?hsa04080+1392 |


---

|  |  |
| --- | --- |
| Pathway | Zn xs inventory |
|  | Zn xs DIN |


---

|  |  |
| --- | --- |
| GO Process | inflammatory response |
|  | long-term memory |
|  | immune response |
|  | parturition |
|  | response to stress |
|  | pregnancy |
|  | signal transduction |
|  | induction of apoptosis by hormones |
|  | synaptic transmission |
|  | learning and/or memory |


---

|  |  |
| --- | --- |
| UniGene | Rn.10349 |
|  | Hs.75294 |
|  | Mm.290689 |


---

|  |  |
| --- | --- |
| Affymetrix Probeset ID | 1369303\_at |
|  | 1457984\_at |
|  | 205629\_s\_at |
|  | 205630\_at |
|  | 40382\_at |
|  | g12803538\_3p\_a\_at |
|  | g4503040\_3p\_at |
|  | M54987\_at |
|  | V00571\_rna1\_at |


---

|  |  |
| --- | --- |
| GO Function | neuropeptide hormone activity |
|  | hormone activity |
|  | adrenocorticotropin-releasing hormone activity |
|  | receptor binding |


---

|  |  |
| --- | --- |
| Nucleotide | AY128673 |
|  | X67661 |
|  | BC002599 |
|  | M54987 |
|  | NM\_205769 |
|  | V00571 |
|  | NM\_031019 |
|  | X55962 |
|  | AK075431 |
|  | S70142 |
|  | BT007453 |
|  | X03036 |
|  | NM\_000756 |
|  | BC011031 |


---

|  |  |
| --- | --- |
| Protein | AAH02599 |
|  | AAH11031 |
|  | P01143 |
|  | CAA23834 |
|  | NP\_112281 |
|  | AAP36121 |
|  | Q8CIT0 |
|  | AAN07905 |
|  | NP\_991338 |
|  | NP\_000747 |
|  | P06850 |
|  | AAA40965 |
|  | CAA26838 |


---

|  |  |
| --- | --- |
| Organism | Mammal |


---

|  |  |
| --- | --- |
| Location | chromosome 8, 8q13 (Homo sapiens) |
|  | chromosome 3, 3 8.0 cM, 3 A2 (Mus musculus) |
|  | chromosome 2, 2q24 (Rattus norvegicus) |
|  | 3 8.0 cM (Mus musculus) |


---

|  |  |
| --- | --- |
